# Coding Conspecific Identity and Motion in the Electric Sense

**DOI:** 10.1371/journal.pcbi.1002564

**Published:** 2012-07-12

**Authors:** Na Yu, Ginette Hupé, Charles Garfinkle, John E. Lewis, André Longtin

**Affiliations:** 1 Department of Physics, University of Ottawa, Ottawa, Ontario, Canada; 2 Department of Biology, University of Ottawa, Ottawa, Ontario, Canada; 3 Department of Cellular and Molecular Medicine, University of Ottawa, Ottawa, Ontario, Canada; Rice University, United States of America

## Abstract

Interactions among animals can result in complex sensory signals containing a variety of socially relevant information, including the number, identity, and relative motion of conspecifics. How the spatiotemporal properties of such evolving naturalistic signals are encoded is a key question in sensory neuroscience. Here, we present results from experiments and modeling that address this issue in the context of the electric sense, which combines the spatial aspects of vision and touch, with the temporal aspects of audition. Wave-type electric fish, such as the brown ghost knifefish, *Apteronotus leptorhynchus*, used in this study, are uniquely identified by the frequency of their electric organ discharge (EOD). Multiple beat frequencies arise from the superposition of the EODs of each fish. We record the natural electrical signals near the skin of a “receiving” fish that are produced by stationary and freely swimming conspecifics. Using spectral analysis, we find that the primary beats, and the secondary beats between them (“beats of beats”), can be greatly influenced by fish swimming; the resulting motion produces low-frequency envelopes that broaden all the beat peaks and reshape the “noise floor”. We assess the consequences of this motion on sensory coding using a model electroreceptor. We show that the primary and secondary beats are encoded in the afferent spike train, but that motion acts to degrade this encoding. We also simulate the response of a realistic population of receptors, and find that it can encode the motion envelope well, primarily due to the receptors with lower firing rates. We discuss the implications of our results for the identification of conspecifics through specific beat frequencies and its possible hindrance by active swimming.

## Author Summary

Effectively processing information from a sensory scene is essential for animal survival. Motion in a sensory scene complicates this task by dynamically modifying signal properties. To address this general issue, we focus on weakly electric fish. Each fish produces a weak electrical carrier signal with a characteristic frequency. Electroreceptors on its skin encode the modulations of this carrier caused by nearby objects and other animals, enabling this fish to thrive in its nocturnal environment. Little is known about how swimming movements influence natural electrosensory scenes, specifically in the context of detection and identification of, and communication with conspecifics. Using recordings involving free-swimming fish, we characterize the amplitude modulations of the carrier signal arising from small groups of fish. The differences between individual frequencies (beats) are prominent features of these signals, with the number of beats reflecting the number of neighbours. We also find that the distance and motion of a free-swimming fish are represented in a slow modulation of the beat at the receiving fish. Modeling shows that these stimulus features can be effectively encoded in the activity of the electroreceptors, but that encoding quality of some features can be degraded by motion, suggesting that active swimming could hinder conspecific identification.

## Introduction

Sensory systems must effectively extract relevant information from an animal's environment. Their ability to encode natural scenes and tease out salient sensory features relies on a range of neural mechanisms, e.g. [1], [2]. In social contexts, individuals generate signals with characteristic temporal and spatial frequencies, and time-varying amplitudes [3]. From these signals, an individual can reconstruct the sensory “social” scene [4] by sorting out the identities, locations and behaviours of its neighbors [5].

**Figure 1:**
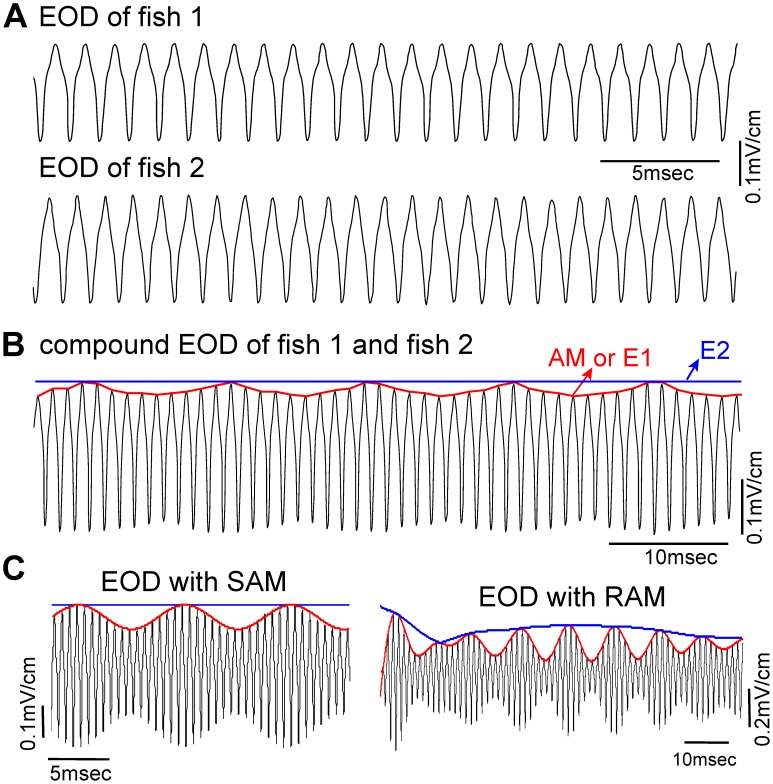
Electric organ discharge (EOD) from weakly electric fish in different situations. (A) Experimental recordings of two isolated individual fish. (B) An example compound EOD recording from two fish in close proximity. The interference of electric fields generated by each fish evokes a time-varying beating amplitude modulation (AM) which is a first order envelope E1 (red trace), as well as a second order envelope E2 (here a flat line, blue trace). (C) The EOD signal with a sinusoidal amplitude modulation (SAM, red trace) or a narrowband random amplitude modulation (RAM, red trace) are common ways to experimentally mimic or computationally simulate electrosensory signals arising from social interactions.

Narrowband signals with slow amplitude modulations, known as envelopes, are a nonlinear signal feature of particular importance for scene analysis in the auditory system [6]–[8], human speech recognition [9], [10], and coding of textures in visual cortex [11]. Envelopes have also been studied in the electric sense [12]–[15]. Weakly electric fish have a submicrosecond-precision neural pacemaker, under behavioural control [16], that produces a weak quasi-sinusoidal dipolar electric organ discharge (EOD). Each animal has its own EOD frequency (EODf) [17]. For example, the species studied here, *Apteronotus leptorhynchus*, has EODfs in the 700–1100 Hz range, with males generally having higher EODfs than females (see Figure 1A for example EOD recordings). These fish sense prey, navigation cues and other animals including conspecifics by encoding amplitude modulations (AMs) of the EOD carrier with the quasi-linear modulation of the mean firing rates of cutaneous electroreceptor afferents [18]–[20].

Two fish in close proximity sense the sum of their electric fields as a time-varying beating AM [17]; the beat frequency is a basic component of electrocommunication [17] (see Figure 1B for example compound EOD with beating AM). In groups of fish, multiple beat frequencies result in “beats of beats” and slow envelopes with narrowband AMs [14]. The spatial aspects of these EOD interactions are less well-understood, though for stati'c fish, the complex electric images of conspecifics have been recently predicted under some conditions [21]. Sinusoidal and narrowband random AMs (SAMs and RAMs, respectively) are typically generated through electrodes to mimic social interactions under experimental conditions (see Figure 1C), and have subsequently led to much insight into the underlying electrosensory processing (e.g. [12], [13], [15], [17]). However, little is known about how movement, resulting in relative changes in distance and orientation, influences the processing of complex AMs in carrier-based senses, such as auditory and electrosensory systems. Signals with SAMs and narrowband RAMs do not have explicit low-frequency power associated with motion. The more natural scenario involves EOD AMs resulting from the motion of a small number of conspecifics [22], which spectrally contain a small discrete set of narrow peaks. A thorough characterization of these natural dynamic signals is necessary to better understand the neural mechanisms required for effective electrosensory processing.

In this study, we first describe the naturalistic AMs and slow envelopes resulting from the relative motion of interacting fish. We contrast the properties of the EOD modulations for static and swimming fish, providing a mathematical model for the associated motion in terms of band-limited random AMs. We then determine the consequences of motion on the neural encoding of number, identity, and movement characteristics of socially interacting conspecifics, by computing what information about the sensory scene is represented in electroreceptor spike trains.

## Results

### Envelope analysis of experimental data

**Figure 2:**
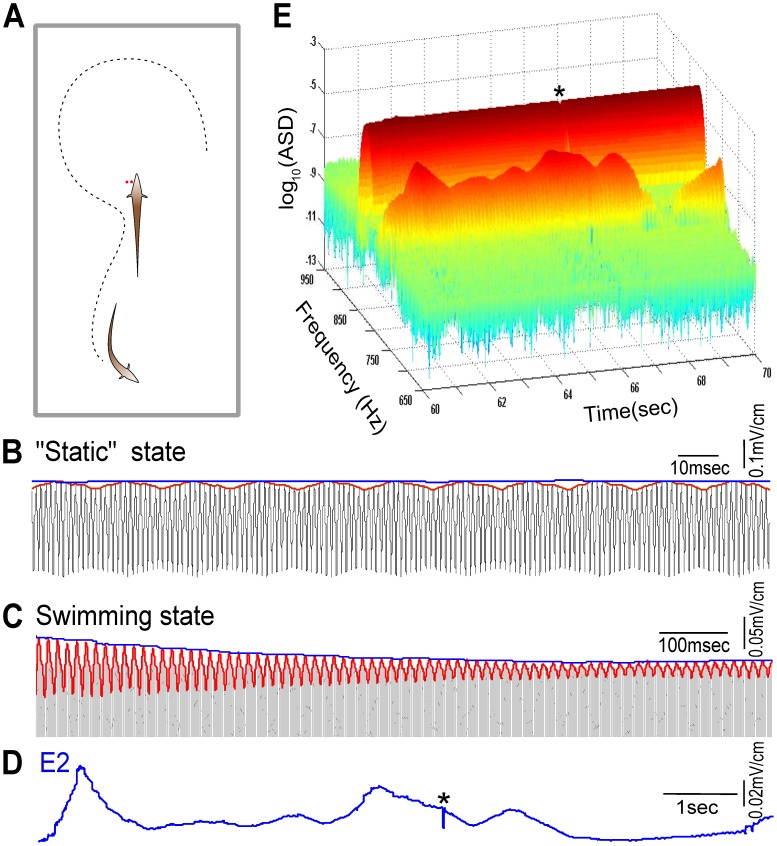
Analysis of experimental recordings. (A) Experimental setup: the fish in the middle of the tank is restrained in a hammock; another one or two fish swim freely. The compound electric organ discharge (EOD) signals due to all fish were recorded through two electrodes (red dots) 1 cm apart and very close to the skin of the head of the restrained “receiving” fish. The line joining the electrodes was perpendicular to the skin in order to measure the normal component of the electric field. (B) Raw electric field (black) recorded from two static fish (both were held in hammocks), and its corresponding first envelope E1 (red) and second envelope E2 (blue). Their EOD frequencies (EODf) are 827 and 763 Hz, respectively. (C) A stretch of data of 1.2 sec long (black, only the positive part is shown) from one restrained fish (EODf at 827 Hz) with one other fish (763 Hz) freely swimming, and the corresponding E1 (red) and E2 (blue). (D) E2 extracted from the recording in (C) over a 10 second duration. The dip around the middle of this trial (marked by “*”, same event as in (E)) indicates a chirp. (E) 3D spectrogram of the data in (D). The amplitude of the spectral density (ASD) of the restrained fish is almost constant at 827 Hz, but the ASD of the freely swimming fish at 763 Hz varies with a very similar pattern as E2 shown in (D).

Since groups of *Apteronotus* in the wild rarely contain more than a few individuals [14], we recorded the signals during interactions of pairs (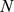 = 8) and triplets (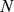 = 4) of fish. The EODfs ranged from 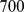 to 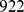 Hz; the beat frequencies (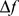), equal to the differences between the EODfs of the interacting fish, ranged from 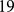 to 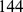 Hz. In each experiment, only one of the fish, named the “receiving fish” and denoted “fish 1”, was restrained. We denote the neighbouring fish as “fish 2” and “fish 3”. The compound EOD signal due to all fish (including fish 1) was recorded through two electrodes locally on one side of fish 1, very close to its head, approximating the signal received by fish 1 near its receptive surface. The other one or two fish swam around freely in the same tank (see Figure 2A and *Materials and Methods*). The amplitude modulation (AM) resulting from the proximity of neighbouring fish is referred to hereafter as the first envelope, E1. The slow envelope of E1 (modulation of the AM) is referred to as the second envelope, E2.

Each individual fish senses its own EOD highly reliably. When a pair of fish are in a “static” state where both are stationary, fish 1 receives a constant stimulus from fish 2 in addition to its own EOD, resulting in a stable periodic E1 at the beat frequency 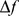 (see Figure 2B). In these conditions, E2, as the envelope of E1, is nearly a constant. In contrast, when fish 2 is allowed to swim freely, both E1 and E2 at fish 1 vary in time (Figure 2C,D). This is a consequence of Coulomb's law, albeit in a complex geometry: shorter distances between the two fish, each of which acts as an oscillating electric dipole, lead to stronger electric current flow caused by the neighbouring fish; larger distances result in a weaker or even undetectable input from the neighbour. Thus, the mean of E2 reflects the average distance between two fish, while the variance of E2 is associated with the pattern of swimming of fish 2 including its bending and turning.

The three-dimensional spectrogram in Figure 2E allows a visualization of the time-varying amplitudes and frequencies of each fish's EOD as experienced by fish 1 for the same segment of experimental data used in Figure 2D. The amplitude of the spectral density (ASD) of fish 1 (around 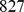 Hz) is very stable in time, but the ASD of fish 2 (around 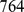 Hz) varies in time as it swims around in the tank, in a manner very similar to the E2 (c.f. Figure 2D). It is worth noting that the occurrence of chirps can also be indicated in E2. A chirp is a communication signal commonly produced during social interactions, and is characterized by a 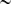
20 msec modulation of the EOD frequency [23]. For example, the fast dip of E2 (“
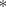
” in Figure 2D) indicates a chirp, which is also seen as a cleft in the ASD (“
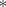
” in Figure 2E) at a time of 65.5 sec. Apart from these brief EODf shifts during a chirp, the EODf did not change over the course of our recordings, and can be assumed constant.

### Mathematical model for the composite EOD signal

*A. leptorhynchus* generates a quasi-sinusoidal EOD, so the superposition of EODs of multiple fish can be well-approximated by a sum of sinusoidal waves at the EOD fundamental frequencies. Since the ASD of the stationary fish 1 is highly reliable, the amplitude of its EOD can be taken as one, without loss of generality; on the other hand, the time-varying ASD from the free-swimming fish 2 is better represented by a stochastic process. Given constant EODfs, the composite EOD signal can be modeled as
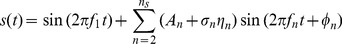
(1)where 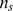 is the group size, 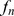 is the EODf of the n-th fish (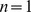 for the stationary fish), and 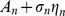 is the stochastic amplitude for the n-th free-swimming fish, with a mean of 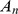 and standard deviation (STD) 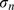. 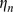 is a stochastic variable with zero mean and unit variance, mimicking the amplitude variations due to movement of the free-swimming fish, and is modeled here for simplicity as an Ornstein-Uhlenbeck process (OUP, or lowpass-filtered Gaussian white noise); the spectral power of an OUP is concentrated in the low-frequency range like the movement itself (see *Materials and Methods*). The phase difference 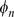 does not affect the spectral components of E1 and E2 and is set to zero; phase may however play an important role in other computations, for example those involved in the jamming avoidance response (JAR) [17].

**Figure 3:**
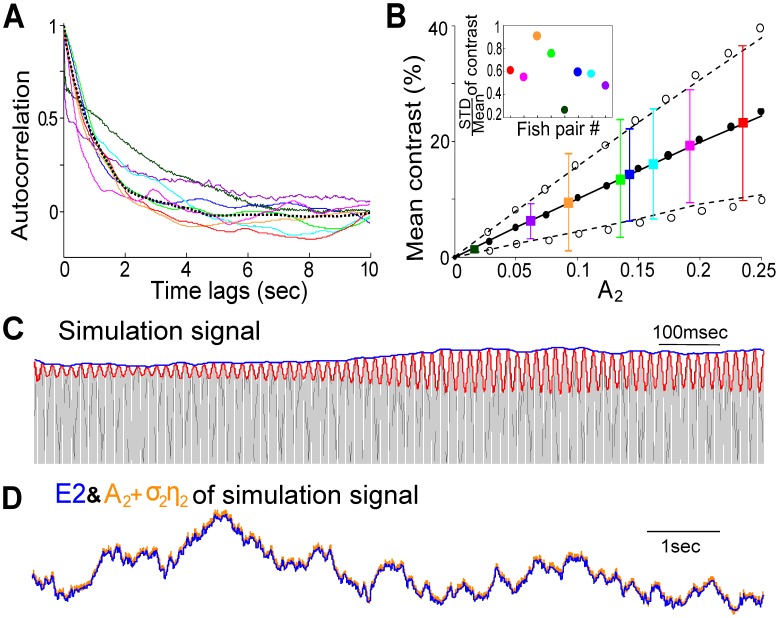
Characterization of the recorded social signal and comparison to the model signal described in Equation (1). (A) Averaged auto-correlation of E2 calculated from five-minute recordings from 8 pairs of fish (each pair labeled by a different color), and from an artificial signal 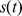 in Equation (1) with 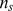 = 2 (dotted line). The Ornstein-Uhlenbeck process (OUP) 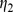 is generated using 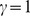 (black dotted curve). (B) Mean contrasts 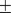 standard deviations (STD) of the raw data from the same 8 pairs using the same color scheme as in (A); numerical results calculated directly from Equation (1) (black lines) and approximate theoretical results (circles, see *Materials and Methods*) showing how the mean contrast (solid black line and solid circles) of the simulation signal 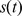 and the mean 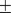 STD contrast with 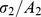 = 0.6 (dashed black lines and open circles) increase with 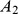; Inset: STD/mean contrast for eight pairs of fish. Note that timeplots in Figure 2C, 2D and 2E, the green curve in Figure 3A and the green data point in Figure 3B are from the same recording of a pair of fish, and it will be used as representative data in the later analysis and figures in the case of two fish. (C) Based on the parameter values provided by this representative data, an example of the artificial signal 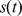 (see Equation (1); black, only the upper part shown here) is shown and its envelopes E1 (red) and E2 (blue) over 1.2 seconds. Its parameter values are 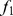 = 827 Hz, 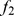 = 763 Hz, 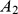 = 0.143, 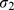 = 0.08, 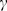 = 1. (D) A comparison between E2 (blue) and the amplitude of the second sinusoidal wave: 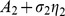 (orange) over 10 seconds.

The OUP is characterized by an exponential autocorrelation function, with a decay time constant that defines its correlation time, 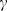 (see *Materials and Methods*). To estimate this correlation time, we compared the autocorrelations of E2 obtained from the experimental trials and the artificial signal 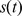 above, for the case of two fish. All autocorrelations of E2 extracted from the natural electric signals recorded separately from pairs of fish exhibit a decaying behaviour (coloured curves in Figure 3A). These experimental curves can be fit very well by E2 of 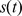 when 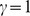 (dotted curve in Figure 3A). This agreement also confirms that the OUP is an appropriate stochastic model for 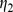. Other parameters 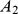, 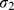 and 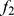 have negligible influence on the autocorrelation as expected from the properties of the OUP and verified by numerical simulation (not shown).

The relative motion of the fish results in a time-varying contrast. The mean and STD of “instantaneous”contrast obtained from the raw data (see *Materials and Methods* for detailed definition) are used to estimate 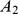 and 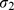, respectively, of the simulation signal, 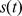, in the case of two fish. Both numerical simulation and theoretical analysis for 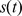 (see Equation (6) in *Materials and Methods*) show that the mean and STD of the contrast of 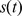 are approximately equal to 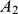 and 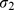, respectively. This is illustrated in Figure 3B which compares the mean contrast from both theory (filled circles) and simulation (solid black line), along with the STD of the contrast (theory: open circles; simulation: dashed black line). Also shown in Figure 3B are the mean contrasts (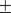
STD) calculated from the recordings of different fish pairs; these values are used as estimates of the corresponding 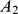 (and 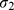) in 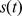. For the five-minute recording with the highest mean contrast of 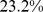 and relatively high STD of 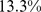 (the fish pair with red data point in Figure 3B), fish 2 was fairly aggressive, making fast approaches sometimes resulting in physical contact. In contrast, for the pair with the lowest mean contrast of 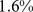 and lowest STD of 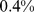 (the fish pair with dark green data point in Figure 3B), fish 2 stayed mainly in the corner of the tank and rarely moved. A similar situation occurred for the pair marked by the purple data point (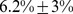), but fish 2 in this trial was slightly more active. This behaviour is also reflected in the autocorrelation times, which are relatively long in these two trials (Figure 3A, dark green and purple curves). The other five pairs of fish exhibited intermediate levels of swimming and approach behaviours (Figure 3B), with mean contrast ranging from 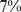 to 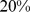 and STD varying from 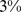 to 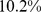. Thus, for the model, we chose the parameter range for 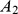 as 0.07 to 0.20, and for 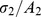 as 0.5 to 0.9.

We can now construct an artificial signal 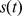 to simulate the signal arising from a real interaction. For instance, Figure 3C shows a realization of 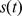 that mimics (statistically) the interaction indicated by the blue fish pair in Figure 3B (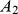 = 0.143 and 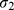 = 0.08), along with its E1 and E2. We also checked the similarity between the calculated E2 and the stochastic amplitude 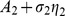 (Figure 3D). The sum of amplitudes and E2 exhibit very good agreement, which confirms again that motion of the fish produces the second envelope E2. The same parameter values will be used in our simulation work below for two fish, unless otherwise stated.

### Spectral properties of the composite EOD signal: implications for electrosensory coding

**Figure 4:**
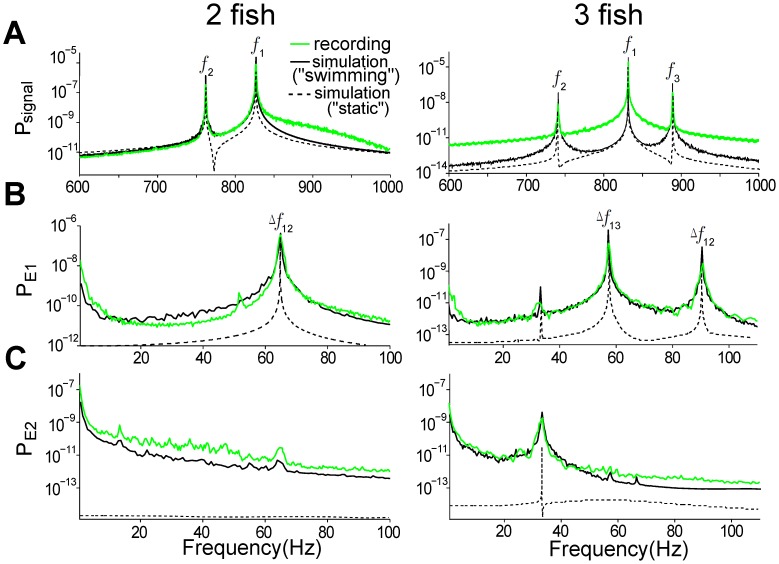
Spectral analysis of sensory signals and their envelopes. Power spectral densities (PSD) of the signal recorded at the receiving fish (A), and its envelopes E1 (B) and E2 (C) from 12-second recordings (green) and the simulated signal (black) for two fish (left column) and three fish (right column). The simulated signal in the swimming state (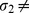
0, black solid curves) is scaled so that its total energy is equal to that of the raw data. The same scaling factor (1
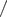
8000) is used to simulate the signal corresponding to the “static” mode (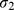 = 0, black dashed curves). Note that the rising power in the low frequency range (0–20 Hz) related to the motion disappears. In the case of two fish, the EODfs of the receiving fish and its neighbour were 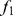 = 827 Hz and 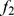 = 763 Hz, respectively, causing a beat frequency of 64 Hz. For three fish, the EODfs of the receiving fish and its two neighbours were 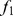 = 831 Hz, 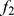 = 740 Hz and 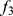 = 889 Hz, respectively, with beat frequencies of 91 Hz and 58 Hz. The secondary beat frequency (i.e. the difference between two beat frequencies, 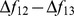) are highlighted by E2. The parameters used for the simulated signal are 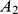 = 0.143, 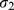 = 0.08 for the two fish case, and 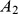 = 0.03, 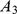 = 0.08, 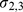 = 0.5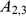 for the three fish case; 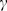 = 1 in both cases.

To quantitatively compare the power spectral densities (PSD) of the simulated signal, 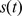, with the raw recordings, 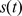 was rescaled to make its total energy equal to that of the experimental data. We consider 12 seconds for two different experimental trials, one from a fish pair and another from a three-fish group (EODfs = [827,763]Hz and [831,740,889]Hz, respectively). The PSDs for the raw signal, as well as for E1 and E2, are plotted side-by-side in Figure 4 (green curves), along with the PSDs for the corresponding simulated signals (swimming state with 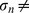
0: black solid curves and static state with 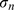 = 0: black dashed curves). The stationary fish 1's EOD is significantly stronger than that of any neighbour, and produces the largest peak in Figure 4A. Thus, the dominant frequencies of E1 are located at the beat frequencies 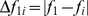, 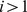, while other beats at 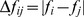, 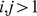 contribute less. Similarly, differences between any two 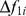
's, referred hereafter as secondary beats, are the prominent spectral components of E2 in groups of three fish (or more). This is evident in the spectral peak of E2 at 33 Hz (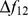
–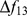 = 91-58) in Figure 4C (right). This clearly describes quantitatively the common notion that electric field modulations at the skin contain spectral information about the number of neighbouring fish as well as their identities (EODf).

Further, at low frequencies (0–20 Hz), the comparison between swimming fish and “static” fish indicates that E1 contains power related to motion (Figure 4B). This power is two or more orders of magnitude less than the power at the beat frequencies. Nevertheless, this contrasts with the narrowband RAMs used in experimental studies [12], [15] to mimic the E1 resulting from the interaction of many static conspecifics; these RAMs have no power at these low frequencies. The E1 motion power (Figure 4B) can also be larger than that of the secondary beats in E1.

Interestingly, the power associated with motion is clearly highlighted by E2 (Figure 4C). This motion produces a decaying spectral floor mainly in the range 0–20 Hz, but extending out beyond 100 Hz over five orders of magnitude or so. The peaks associated with the secondary beats ride on top of this floor, with very low power for the chosen EOD parameters. Neural circuitry specialized in extracting information from slow E2 envelopes [7], [11], [12] could do so using the lower frequency structure; in this context, the E2 floor would be considered a signal. Alternatively, this E2 power could obscure other potentially significant envelope signals in the same range, and this motion-induced spectral floor would act as a noise floor. Finally, Figure 4 shows that there is very good agreement, both qualitative and quantitative, between the spectral features of the experimental and simulated signals, providing further support for our model.

**Figure 5:**
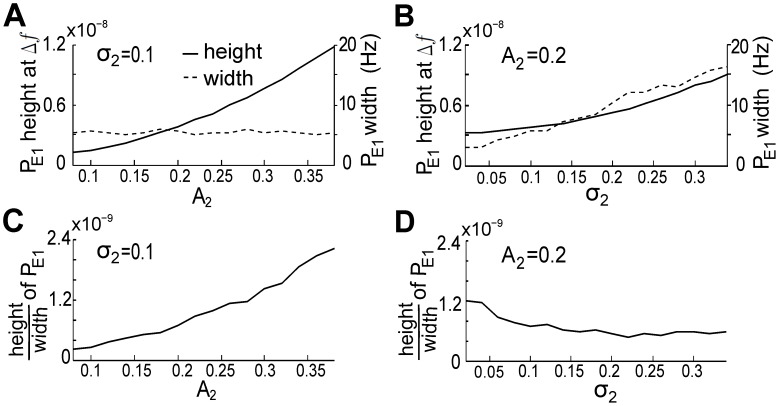
The mean amplitude and standard deviation of the EOD of the swimming fish influence the spectral characteristics of E1. (A, B) The height (solid line), width (measured at 3
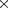
10^−9^, dashed line) and (C, D) resolution (defined as the ratio of height to width) of the peak of 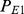 at the beat frequency, 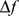, with increasing 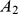 and fixed 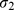 = 0.1 (left column), or increasing 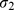 and fixed 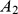 = 0.2 (right column). Increasing 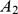 improves this resolution, whereas the increases in 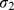 decreases this resolution. Other parameters here are the same as those in the case of two fish in Figure 3. 50 independent OU process realizations were used to produce theses averaged plots.

Electric fish recognize the EODf of conspecifics through the beat frequencies [17]. Therefore, higher spectral peaks or narrower peak width (PSD of E1) at beat frequencies should improve the ability of fish 1 to encode the beat frequencies. To investigate how inter-fish distance and motion can influence this encoding, we define the spectral resolution of beat frequencies in E1 as the ratio between the height and width of the corresponding spectral peaks (see Figure 4B). 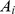 and 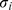, which relate respectively to the inverse of the distance between two fish and the movement variation of neighboring fish, are important factors for quantifying E1. A group of two fish, the simplest and most common group for *A. leptorhynchus*, with the same EODfs as in Figure 4 was taken as an example. We computed the average height and width of the PSD peaks centered at beat frequencies for different combinations of 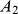 and 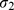 and plotted the simulation results in Figure 5. The peak width is measured at a power of 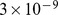 (slightly above the “noise floor”), because the increment of spectral peak width is more sensitive to 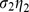 at this value. Figure 5A clearly demonstrates that when 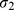 is fixed at 0.1, a larger 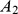 results in an increased peak height (solid line), while the peak width (dotted line) barely changes. Therefore, shorter distances between two fish increase the resolution of the beat frequency (see Figure 5C). Figure 5B shows that, for fixed 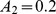 and increasing 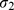, both width and height increase [24], but at different rates. The result is that the ratio of height to width decreases with increasing 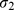 (by about 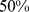 over the range tested, see Figure 5D), indicating that a larger swim variance of fish 2 reduces the spectral resolution of the beat frequency. A comparison of the rates of changes in Figure 5C and 5D indicates that the resolution of the beat frequency is more sensitive to 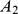 than 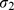.

**Figure 6:**
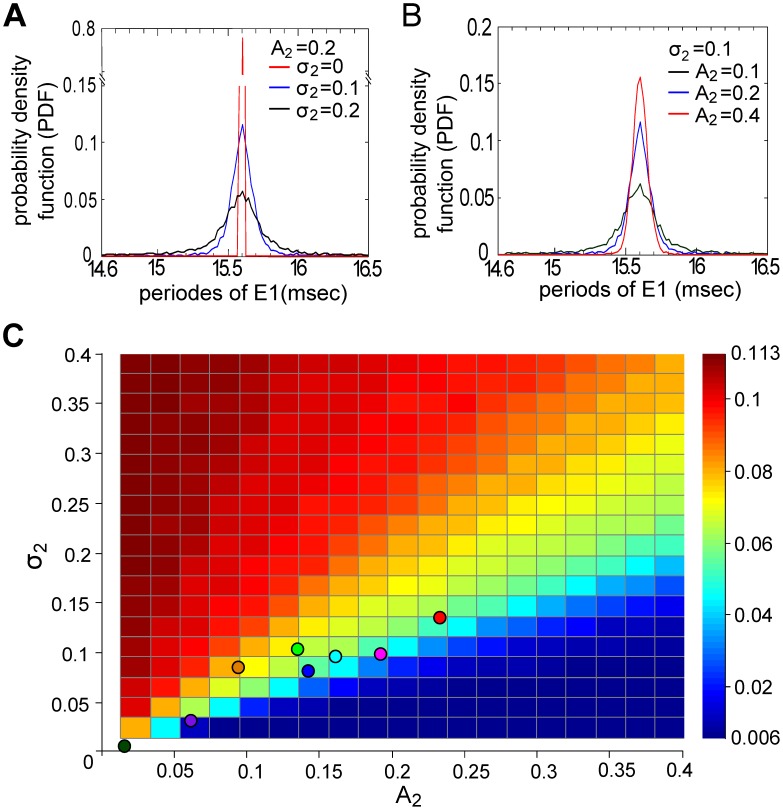
The fluctuations in the period of E1 received by the stationary fish vary with the motion of the neighboring fish. Probability density function (PDF) of the periods of E1 when 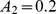 and 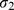 changes (A), or when 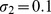 and 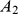 changes (B). The binwidth is 0.02 msec. A larger 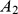 produces more periods at precisely 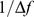; a higher 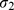 disperses the periods over a broader time interval. (C) The coefficient of variation (CV, the ratio between STD and mean) of the periods of E1 increases with 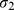, but decreases with 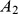. Combinations of 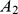 and 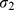 corresponding to experimental trials are marked by dots with the same color scheme as in Figure 2B.

The influences of 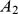 and 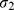 on E1 can also be observed in the time domain, via the behavior of successive periods of the E1 waveform. For the current example, the probability density function (PDF) of the E1 period shows a peak at 15.6 msec (i.e. beat period of 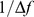 = 1/64 Hz) when 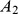 = 0.2 and 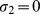 (Figure 6A; red line); a larger 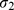 introduces more jitter around this beat period. On the other hand, a larger 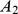 reduces fluctuations of the beat period (Figure 6B). We quantify these effects using the coefficient of variation (CV), defined as STD divided by the mean of the periods of the E1 waveform (Figure 6C). Over the parameter range shown, increasing 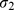 leads to a larger CV, whereas increasing 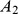 decreases CV. Interestingly, combinations of 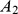 and 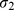 corresponding to the experimental trials (plotted as filled circles in Figure 6C, colored as in Figs. 2 and 3) show a systematic relationship, suggesting that the fish do not vary distance and motion independently under the conditions tested (whether or not this is a general feature of social interactions will be determined in future studies). In summary, both types of analysis suggest that increasing inter-fish distance (decreased 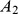) and increased motion (increased 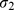) lead to a degradation in the quality of the E1 signal with respect to the beat frequencies. In the next section, we assess the impact of 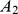 and 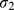 at the level of sensory encoding by considering the responses of model electroreceptors (P-units) to these same signals.

### Electroreceptor responses to neighbouring fish

P-unit electroreceptors are the first processing site in the electrosensory pathway, encoding information contained in the transdermal voltage fluctuations. Using artificial SAM and RAM-type signals, P-units have been shown to encode the time-varying raw electrical signal into instantaneous changes in their stochastic firing rate [25], [26]. These changes track (almost linearly) the AM represented by E1 in those studies (except at higher stimulus contrasts where nonlinear effects are involved [13], [15], [27]). The leaky integrate-and-fire model with dynamical threshold (LIFDT, see *Material and Methods*) has been shown to capture most essential features of the spiking dynamics of P-unit afferents [13], [28]. Therefore, to provide insight into electrosensory coding during natural interactions, we describe the response of this P-unit model to the composite EOD signals described in the previous section.

**Figure 7:**
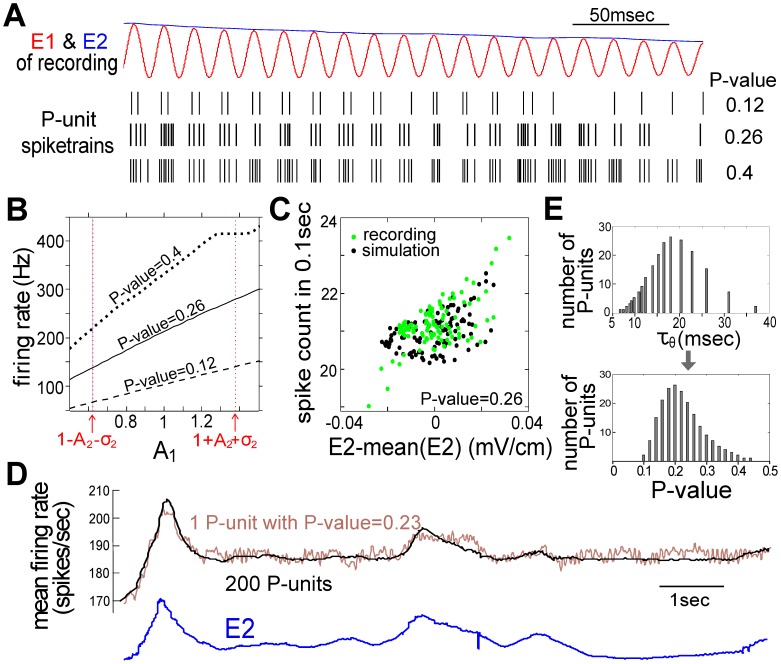
The response of electroreceptors (P-units) to the motion stimuli. (A) Three examples of spike trains generated by the P-unit model (P-value = 0.12, 0.26 and 0.4) in response to the sensory input of the recording plotted in Figure 2C with E1 (red) and E2 (blue). (B) With 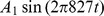 as input (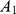 is constant), the firing rate of the P-unit increases with increasing 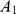. The range of the envelope, 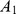, is mainly 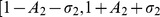] as indicated in equation (6) in *Material and Methods*. (C) Within a time window of 0.1 second, the number of spikes increases with increasing E2. These data are extracted from recordings (as in Figure 2D) and simulations (as in Figure 3D). (D) Mean time-dependent firing rate (black trace) obtained from 200 independent P-units, each with its own internal noise and baseline firing rate set by the parameter 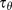 (see panel E), exhibits a time-varying curve similar to E2 (blue trace, as in Figure 2D) of the recording that was used as input to all 200 P-units. The colored trace is an example of the time-varying firing rate of a single P-unit with a P-value of 0.23. (E) Varying 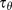 in the P-unit model (equation (7) in *Material and Methods*) according to the density shown on the top generated a good approximation to the experimentally observed heterogeneity in P-values shown on the bottom (the latter being well-fitted to a log-normal density in [29]). It also leads to a good agreement with the experimental observed envelop-coding ability of P-units in [15] (see Figure S1).

Figure 7A shows three example spike trains 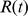 from the model P-units with different P-values (see *Material and Methods*) in response to the recording from Figure 2C (with E2, spectrogram and PSD shown in Figure 2D, E and Figure 4A, respectively). These spike trains clearly show that the instantaneous firing rate increases with increasing E1. To investigate the envelope-output transfer function of a P-unit, we use the simplest signal 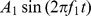 (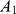 is constant) as the input instead of 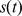 in Equation (1). According to Equation (6), the motion of fish 2 can be seen as fluctuations in the envelope 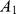 mainly in the range of 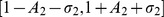. Previous studies have shown that P-units can exhibit firing rate saturation with time-varying E1 [15], [29]. Here Figure 7B demonstrates that, within the range of interest, the output firing rate is basically proportional to 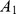; P-units with larger P-values simply encode the EOD fluctuations into modulations of a higher baseline firing rate. Figure 7C demonstrates the E2-output transfer function, where the spike counts within 0.1 second increase with increasing E2 in the same time window. This suggests that the motion of neighboring fish varies the firing rate of P-units of fish 1.

**Figure 8:**
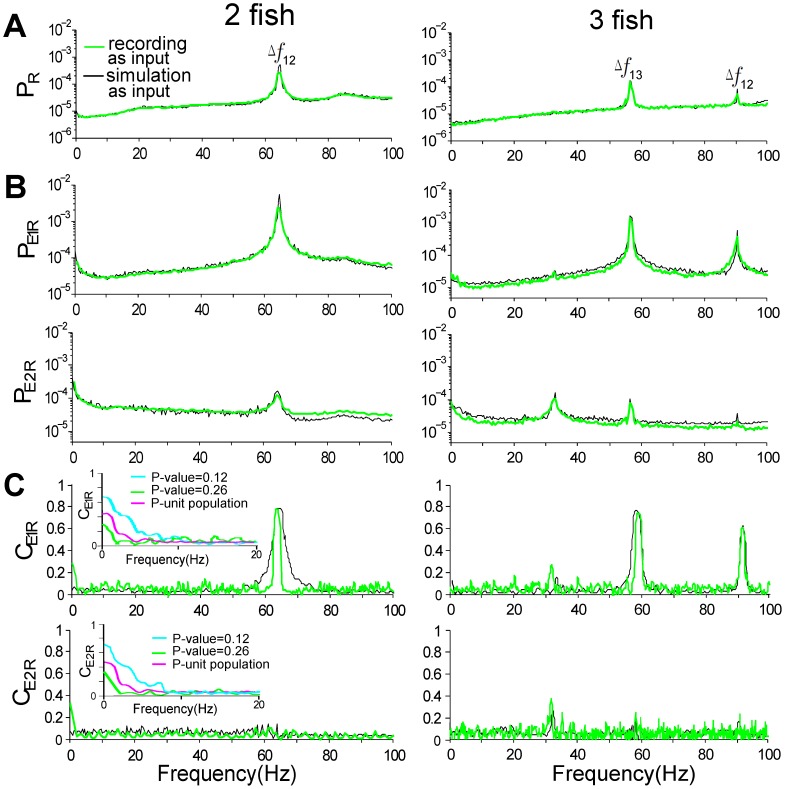
The information extracted from spike trains of P-units. (A) Averaged PSD of the simulated P-unit response 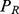. (B) Cross-spectra 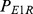 between E1 and the P-unit response, and cross-spectra 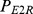 between E2 and the P-unit response. (C) Coherence 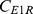 between E1 and the P-unit response, and coherence 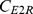 between E2 and the P-unit response; we compare the coherence functions of P-units with different P-values over 0–20 Hz in the inset, showing that P-units with low P-values can better encode motion-related information than those with high P-values. Results are shown for two fish (left column) and three fish (right column) and P-unit model with P-value of 0.26 (green and black curves), P-value of 0.12 (cyan curves) and a population of 200 P-units with variable P-values as shown in Figure 7E (magenta curves). The recordings in Figure 3 were used as input to the P-unit model (green, cyan, magenta traces); the same parameter values in Figure 3 were used for simulation input (black traces).

This can be clearly demonstrated by looking at the time-varying firing rate calculated from a heterogeneous population of P-units in Figure 7D. Each electroreceptor has its characteristic P-value, and across receptors, the P-values form a log-normal distribution with a mean value at 0.26 (see [29] and Figure 7E). We calculated the mean firing rate with a time window longer than 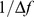 (e.g. 0.1 second) using models of 200 P-units with such distributed P-values. We also computed the time-varying firing rate of a single P-unit with a P-value equal to 0.23 for comparison. By comparing E2 with the firing rate curves obtained from the P-unit population and single P-unit in Figure 7D, we can conclude that the population encodes the motion of the neighboring fish better than individual P-units with average to large P-values. Note however that the single unit already encodes it quite well on its own, at least over this frequency range. Also note that the raw input signals here do not have direct power at the beat frequencies, and so the extraction of the beat frequencies must involve a nonlinear operation. This nonlinearity is implemented in our analysis by the Hilbert transform (HT, see *Materials and Methods*), allowing us to obtain the E1 of the raw signal (Figure 4B). However, implementation by the P-unit model involves the spike threshold (and possibly other) nonlinearities [12], [13], [15]. The P-unit plays a role similar to that of the HT to extract E1 and eliminate the EODfs. The power spectrum of P-unit spike train in Figure 8A indicates peaks at beat frequencies (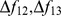) which are not presented in the PSD of input signal but are in the PSD of E1. Further, the peaks at 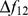 in E1 are strongly correlated to the spike train (see the cross spectral density 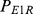 between E1 and the P-unit response in Figure 8B). However, 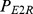 reveals that these peaks are much less correlated to E2 (Figure 8B).

The coherence was then used to estimate the linearity of the encoding of E1 and E2 by the output 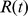 of the P-unit model (see *Materials and Methods*). For P-units with a P-value of 0.26, the E1-R coherence, 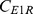, has a peak at 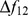 (Figure 8C), suggesting that P-units can efficiently encode this beat frequency. However, the very low E2-R coherence, 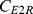, implies that most individual P-units do not linearly represent information about slow envelopes associated with natural motion (Figure 8C), except perhaps at the very low frequencies where a slight rise is seen. However, the coding of motion-related information can be improved for P-units with low P-values (e.g. 0.12 marked by green dashed curve in Figure 8C). Further, as numerous P-units participate in the processing of sensory information, a population code could relay motion-related information embedded in E2 to downstream electrosensory neurons (see insets of Figure 8C). For the case of three fish (Figure 8, right panels), the same features hold qualitatively (even the low frequency motion - not shown). In addition, the slower secondary beat frequency is clearly revealed by the 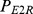 response function (Figure 8B), as was seen for the raw signal in Figure 4C. 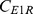 now has a large peak at both main beat frequencies, and a very small peak at the secondary beat frequency. 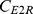 again emphasizes the slower secondary beat.

**Figure 9:**
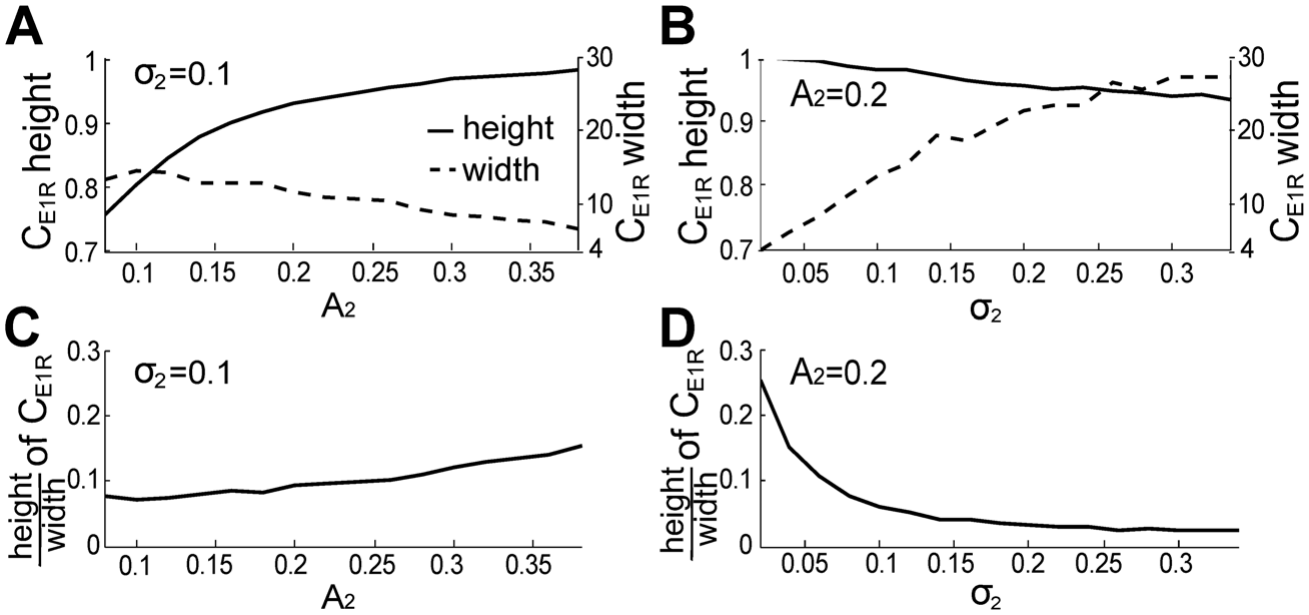
A higher mean amplitude or lower standard deviation of the EOD of the swimming fish facilitates estimation of beat frequency at the receptor level. (A) The height (solid line) of the peak of 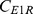 at the beat frequency, 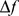, and the width (dashed line) of this peak at the coherence value of 0.15 increase and slightly decrease, respectively, with 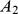 (with fixed 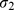 = 0.1). However (B), the above height and width slightly decrease and strongly increase, respectively, with 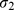 (with fixed 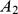 = 0.2). Therefore (C), the height-to-width ratio of this 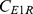 peak slightly increases with 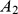, while (D) it decreases rapidly with 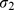. The curves in this figure and next figure are the average results over five artificial signals with 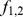 = [827,737], [827,760], [827,792], [827,704], [740,807]Hz; 50 independent OU processes 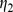 were used to calculate the average for each artificial signal.

We now describe the influence of 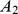 and 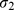 on E1-R coherence at the beat frequency for two interacting fish. Similar to our evaluation of the PSD peaks, we measure the height and width of the coherence peaks to quantify coding quality. Figure 9A shows that the maximum height of the 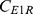 peak at the beat frequency increases with 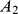, and the width (measured at a coherence of 0.15, slightly above “noise floor”) slowly decreases with 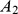. This leads to an increasing height-to-width ratio with A2, and thus could improve the accuracy of beat frequency estimation in the hindbrain (Figure 9C). Since 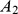 varies inversely with inter-fish distance, this confirms that at the receptor level, shorter distances between two fish enhance their ability to detect each other via the beat. On the contrary, increasing 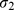 (akin to increasing the strength of swimming variation) enlarges the 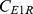 peak width and decreases the height (Figure 9B); consequently the height-to-width ratio drops with 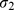, i.e. when swimming is more erratic or less confined (Figure 9D). This implies that rapid changes in distance between two fish could blur the sensing of the other through the beat frequency. The same conclusion can be obtained using the width measurements at half max (not shown). With multiple fish, this blurring would have even more impact if beat frequencies were close. Thus motion, through degradation of the P-unit encoding of the beat frequencies, could be actively used as a form of crypsis, decreasing identification by conspecifics.

**Figure 10:**
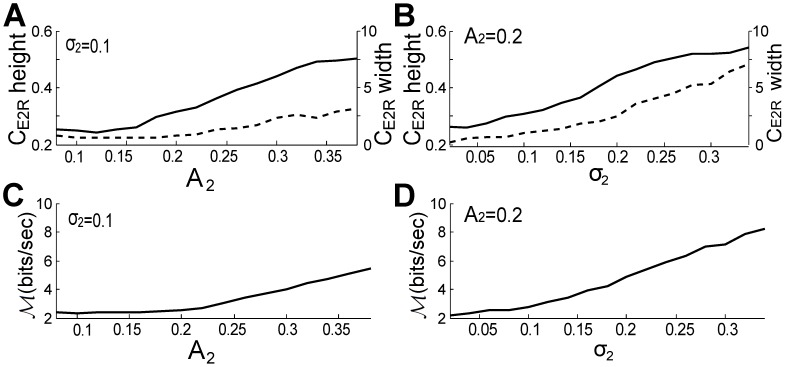
Increasing 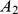 or 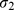 enhances the motion-related information gained by the electroreceptors in the case of two fish. (A–B) The maximum (solid line) of 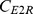 over the 0–20 Hz range and the width (dashed line, measured at 0.15) increase, with 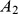 (and fixed 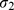 = 0.1), as well as with 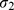 (and fixed 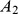 = 0.2). Higher mutual information (MI) rate, 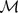, could be obtained over 0–20 Hz with increasing 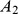 (C) and 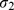 (D). The numerical method to obtain the height and width of 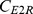 is described in Figure 9.

Considering that most of the motion power is concentrated over the frequency range of 0–20 Hz in E2 (also see [30], [31]), the mean peak coherence of 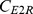 over 0–20 Hz was plotted to examine the information encoded from E2. In Figure 10A and B, the peak height and width of 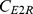 increase with both 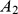 and 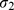 over 0–20 Hz. Similarly, the mutual information rate over 0–20 Hz also increases with both parameters (Figure 10C,D). These results show that electroreceptors encode motion of conspecifics increasingly well for smaller inter-fish distances and increased relative movement. Thus, it appears that when a fish increases movements towards a conspecific, there may be a trade-off between improved encoding of motion (as a signal or a noise, as mentioned above) and degraded identification, which are coded by E2 and E1, respectively.

## Discussion

Our study describes the naturalistic signals generated by relative motion among small groups of weakly electric fish. The analysis of the raw signals and the simulated responses of primary electroreceptor afferents show that these signals contain important cues for the identification of individuals and their behaviour. This information is available from the spectral properties of the first and second envelopes (E1 and E2) of the composite electrical signal, which relate to the beat frequencies (E1) and the secondary beats and relative motion patterns (E2).

The phenomenological model for motion fitted our data very well, and its parameters 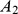 and 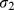 are directly related to the contrast mean and contrast STD of the experimental recordings (Figure 3B). Further, our experiments revealed a proportional relationship between the STD and mean of the contrast (and thus between 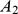 and 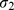; see Figure 6). However, we can not infer that this relationship is universal across all experimental and social contexts. The possible context-dependence and behavioural significance of the relationship will be explored in future studies. In addition, the relationship between model parameters 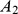 and 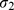 and behavioural measures is not entirely clear. While the mean contrast 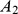 is inversely related to the mean distance separating the fish, it is also influenced by the complex interactions between fish bodies [21]. We also note that 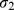 is related to motion (variations in swimming). This relationship is complex and is influenced not only by changes in inter-fish distance, but also by turning and bending. A thorough characterization of the physical bases of 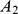 and 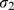 is beyond the scope of this study and will be pursued in subsequent work.

Our experimental and modeling work shows that movements of neighbouring fish generate power in the first envelope E1 that is small relative to the power in the beat (AM of the sum of EODs; Figure 4B). But the movements produce relatively more power in the second envelope E2 (envelope of the AM; Figure 4C), especially below 10 Hz. Our model reveals that the peak resolution for the beats in E1 increases slightly over a range of amplitudes 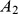, i.e. of contrasts (Figure 9C), but decreases strongly with motion stochasticity (Figure 9D). It also reveals that the encoding of E2, while no longer representing beats, is proportional to the mean amplitude of the neighbouring EOD (Figure 10A,C), which is inversely proportional to inter-fish distance. The encoding of E2 is also proportional to the variance of the motion (Figure 10B,D). E2 also highlights the secondary beats between the primary beats. As the electrosensory system can extract the envelope post-synaptically to the electroreceptors [12], such envelope information about motion and secondary beats can be readily relayed to midbrain electrosensory regions. Our results imply that this structure has access to both the beats, the secondary beats and motion information, which can in principle feed the directional selectivity circuitry [32].

Further, our analysis suggests that P-units effectively encode beat frequencies, but single P-units with normal-to-large P-values can not encode motion information well over a range of normal contrasts (Figure 8C). This is consistent with previous modeling [13] and experimental work [15] where E2 obtained from narrowband RAMs could be represented by P-unit activity only for large contrasts, and otherwise the transmission of E2 to higher-order cells relies on a parallel pathway via interneurons [12]. Another experimental study reported a tracking between mean firing rate of P-units and a low-frequency 0–4 Hz RAM [29]; taken together with our observation that P-unit mean firing rate varies with E2 (Figure 7), this suggests that a population code might instead be involved in encoding E2.

These fish can transform spatial information about the motion of other fish into a temporal signal with a second envelope. The amplitudes of the EODs reflect the distances of fish 1 to its neighbours, and are clearly reflected in the height of the beat peaks in E1, as well as the mean of E2. Therefore E2 may play an important role in electrolocating conspecifics. It remains to be seen whether E2 improves stimulus localization, as can occur for static auditory sources [6].

The identity of conspecifics, given by their individual EODfs, is well represented by beat peaks in E1, especially at short distances (large 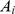). However, for dynamic swimming (larger 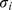), these peaks broaden, and the sensory system may no longer be able to differentiate different beats that are close in frequency. Animals use various forms of camouflage and other behaviours to avoid predators. Non-visual crypsis has been reported in auditory, olfactory, and electrosensory systems in recent years [33]. Electric fish have a high risk of being detected by electroreceptive predators, and therefore may have to take extraordinary measures to protect themselves. The pulse-type fish *Brachyhypopomus* may use “signal cloaking” by shifting the spectrum of its EOD pulse to a less detectable high-frequency range [34]. Other species of electric fish must use other strategies to avoid detection. Figure 9 predicts that identification (via EODf) declines with increasing 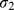, suggesting that fast motion (e.g. back-and-forth swimming, as well as rapid bending, turning or spinning) could be another implementation of non-visual crypsis. The well-described behaviours requiring EODf estimation (such as the jamming-avoidance response, JAR [17]) make wave-type electric fish an attractive model in which to test this intriguing hypothesis.

Our study also points to a novel method of synthesizing more natural mimics of other fish in the laboratory. The established approach uses a SAM modulation of a restrained fish's EOD, and thus mimics a static conspecific. This actually leads to additional frequency components of the EOD that are not present naturally. The model signal presented here could be used to mimic swimming conspecifics, applied either locally, or globally using the usual configuration of two electrodes straddling the animal, or with a method that better preserves ipsilateral and contralateral contrasts and polarities [21].

The results demonstrated in this study involved one artificially restrained fish. The more natural situation is of course that in which all fish are free to swim. The question then arises as to what influence self-motion has on the results of our analysis. This means that the amplitude 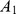 in our model would not be fixed but would vary with self-motion, and the P-units would encode the associated potential excursions. This is known from e.g. tail movements [35], [36]. While a full analysis of this problem must rely on actual measurements and involve field simulations, we can speculate that self-movement will likely have an impact on any identification and crypsis strategy. We note however that some body movements (such as tail bending) are known to be cancelled by plasticity at the pyramidal cell level [36]. This may mitigate self-motion signals, and emphasize beats and motion signals due to the relative motion of other conspecifics.

The proximity of the fish in our experiments resulted in chirping behaviour, and the features of E1 and E2 that triggered such communication will be explored elsewhere. This nonetheless raises the possibility that certain patterns of E1 and E2 lead to changes in the EODfs of the interacting fish over longer time courses, as they may avoid certain low frequencies that interfere with e.g. prey stimuli [14], [37]. Likewise the fish may engage in the JAR, which are predictable in static SAM-type mimics of three interacting fish in a related species [38]. The interplay of jamming, chirping and movement can be used in experiments to understand more properties of the primary afferents, which exhibit non-trivial responses to AMs and their slow envelopes. It would also be interesting to eventually relate these findings to those on hydrodynamic cues for the lateral line detection system [39]. Future work should also consider the phase variations of E1 and E2 across fish 1 as neighbours move, and on which the JAR relies. Our study highlights important information available for the analysis of such complex social sensory scenes.

## Materials and Methods

### Ethics statement

All experimental protocols were approved by the University of Ottawa Animal Care Committee (BL-229).

### Experiments

*A. leptorhynchus* were obtained from a tropcial fish supplier and housed in 115 L flow-though community tanks maintained at 26–29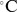 with a conductivity of 200–250 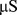. Fish were kept on a 12h∶12h light∶dark cycle, and fed frozen bloodworms 3 days per week. Five fish were chosen randomly for our analyses and isolated in 20 L tanks. Recordings were performed in an experimental tank measuring 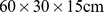 in length-width-depth. During the trials, the restrained fish (fish 1) was placed in a hand-sewn tulle hammock, closed along the top with a strip of Velcro and suspended in the middle of the experimental tank. The top of the hammock was positioned about 1 cm below the water surface. Fish 1 was unable to turn or swim, and tended to remain quite still while in the hammock. Depending on the trial, one (two fish experiment) or two (three-fish experiment) other fish were added to the tank, and allowed to swim freely around the centrally positioned fish 1.

To record the natural inputs resulting from interacting conspecifics, a pair of Teflon-coated silver recording electrodes (diameter: 0.38 mm; WPI, Inc., Sarasota, FL, USA) were positioned adjacent to the head of fish 1 just anterior to the operculum. The exposed electrode tips were 1 cm apart, and oriented perpendicular to the axis of the restrained fish (Figure 2A) to measure the component of the electric field normal to the skin. A grounded Teflon-coated electrode (insulated to the tip) was attached to one corner of the test tank. The electrical signals were amplified using an AM Systems model 1700 (Carlsborg, WA, USA) differential amplifier (100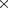 amplification, low-frequency cut-off of 10 Hz, high frequency cut-off of 5 kHz) and sampled at 100 kHz using a dSpace Inc (Wicom, MI, USA) 1011 data acquisition board and dSpace Control Desk software. During the dark stage of the daily cycle, a randomly chosen fish was isolated, and its EOD was recorded in isolation. A second, free-swimming fish was added to the experimental tank, and a five-minute recording began. After five minutes, the free-swimming fish was removed and returned to its home tank. The water heater was removed during the 5-minute interactions and replaced afterwards to maintain temperature at 26–27

C. Recordings from eight pairs and four triads of interacting fish were obtained, without controlling for sex. Fish were identified based upon their anatomical differences and EODfs.

All data analyses and numerical simulations were carried out in MATLAB (The MathWorks, Inc., Natick, MA, USA).

### Envelope extraction

The envelope of a real-valued oscillatory process 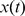 is defined by 
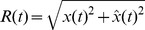
 where 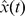 is in quadrature with 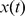. 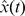 is commonly obtained using the Hilbert transform (HT) [40] defined by
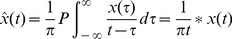
(2)with P denotes the Cauchy principal value and * denotes convolution. Thus one can create an analytic signal 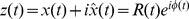, and obtain an instantaneous amplitude 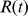 and instantaneous phase 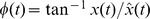 of the raw signal. 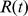 and 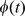 have a clear physical meaning when the amplitude evolves on a slow time scale compared to the fast phase [40]. 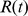 and 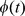 extracted from an EOD recording that consists of multiple harmonic frequency components of the EOD share a remnant spectral peak at the EODf. A low pass filter (LPF) was used following the HT operation to eliminate the EODf and obtain the correct first envelope E1. Its cut-off frequency was set to 200 Hz. A HT was implemented to extract the second slower E2 envelope segments from E1 over every 0.1-second time window; these segments were assembled end to end to produce E2. E1 and E2 obtained above were also compared with the direct envelope extraction (i.e. connecting the successive peak points of EOD cycles or E1 oscillatory curves [41]). The PSD profiles and time series of E1 and E2 calculated from both ways are very similar.

### Ornstein-Uhlenbeck Process (OUP) for motion

The OUP, 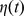, a simple form of lowpass-filtered Gaussian white noise, is used here to model the stochastic EOD amplitude caused by a free-swimming fish at fish 1. It is the solution of
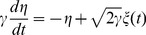
(3)where 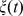 = 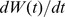 is Gaussian white noise of zero mean and autocorrelation 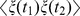 = 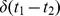, and 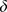 is the Dirac delta function. Brackets denote average over the Gaussian ensemble. 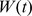 is Brownian motion, whose increments 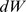 are taken from a zero-mean Gaussian density with a variance of 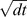. The OUP has zero mean and is exponentially correlated: 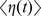 = 0 and 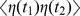 = exp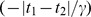; it has unit variance. The correlation time is 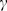; the larger it is, the slower the exponential decay of the autocorrelation, and the slower and smoother the fluctuations in time are.

### Autocorrelation

The autocorrelation function 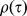 quantifies the average linear correlation between successive points in a time series 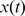, as a function of the temporal lag 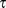 between these points:
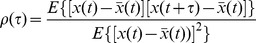
(4)where 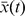 is the mean of 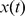 and E denotes the expected value. For the envelopes, E2, 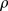 was calculated from a 5-minute uninterrupted recording for one pair of fish by dividing the record into 15-second segments. The autocorrelations over each segment were averaged and plotted as one coloured curve in Figure 3A.

### Mean and STD of contrast

An EOD carrier with frequency 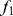 and a random amplitude modulation (RAM) is denoted as 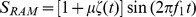 where 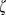 is a random process. Contrast is defined as (STD of AM)
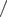
(mean of AM) = 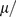
1 = 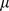, i.e. it is the coefficient of variation (CV) of the AM. An EOD carrier with sinusoidal amplitude modulation (SAM) is denoted as 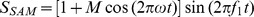. Its contrast is (STD of AM)
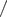
(mean AM) = M/1 = M.

According to the HT defined above or the method in [42], the AM (or E1) of our model signal 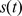 in Equation (1) with 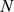 = 2 can be expressed as
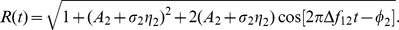
It can also be rewritten as
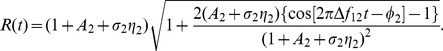



We can use the Taylor expansion with 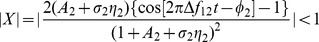 to get an approximation for the AM:
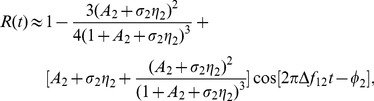
(5)where higher order terms with frequencies at harmonics of 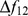 are neglected, a procedure similar to LPF used in the numerical approach described in the section “Envelope extraction”. Because 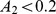 and 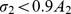 (as seen in Figure 3B), 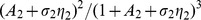 is a small perturbation and can be approximated by 0. Thus we have
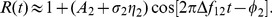
(6)The AM of 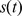 thus combines the RAM and SAM. If we regard this AM as a special version of a SAM, its contrast is approximately equal to 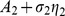 because higher order terms are neglected. 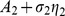 contains fluctuations introduced by 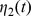, therefore we have a mean contrast 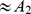 and STD 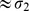. For the contrast calculation of the experimental data, we extracted the AM, then collected all its highest and lowest points of AM and calculated an “instantaneous” contrast = 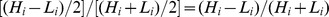, i.e. the half-difference between a highest point 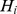 and the lowest point closest on its right 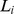, divided by the average of 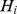 and 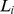. Mean contrast and STD derived from Equation (6) are consistent with this definition by taking 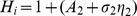 and 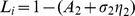. The mean and STD of these instantaneous contrasts for each pair of fish are plotted in Figure 3B.

### P-unit model

The linear integrate-and-fire model with dynamic threshold (LIFDT) used to simulate P-unit afferents is written as
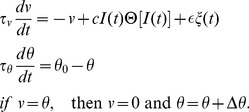
(7)

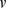
 represents the transmembrane potential measured from its resting level; 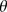 is a dynamical threshold incremented by a fixed amount 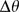 every time P-unit fires; 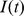 is the input to P-units, here the experimental recording or the simulation signal 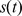 used as the input. 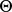 is the Heaviside function that accounts for the fact that many receptors rectify a periodic forcing [43]. 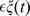 mimics intrinsic noise, where 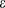 is the noise intensity and 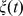 is Gaussian white noise with zero mean (different from that used to generate the OUP). Parameter values are 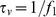, 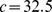, 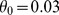, 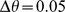, 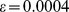, and the time step is 0.01 ms. A P-unit fires when 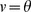 after which the voltage is reset to zero. The firing times 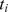 generate a binary spike train 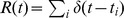.

The P-value is the characteristic parameter of a given receptor [29], [44]–[46]. It is calculated as the baseline firing rate (i.e. with the EOD of fish 1 alone and no AM) of P-type afferents, divided by the EODf. Because P-values follow a log-normal distribution and ranges from 0.1 to 0.6 with a mean value at 0.26 [29], we vary 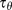 to obtain different P-values as shown in the densities of Figure 7E.

### Coherence

This statistic is used to measure the linear relationship between the frequency components of two signals, 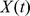 and 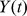. It can be seen as a signal-to-noise ratio, and reflects a lower bound on the mutual information (see below) between input and output. It is defined as
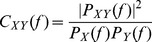
(8)where 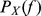, 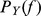 are, respectively, the auto-spectral densities of 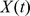 and 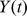, and 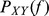 is the cross-spectral density of 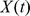 and 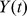. As 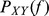 is the Fourier transform of the cross-correlation between 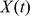 and 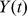, coherence can be regarded as a correlation coefficient in the frequency domain, ranging at each frequency between 0 (no linear correlation) and 1 (perfect linear correlation).

### Mutual information (MI)

Mutual information (MI) quantifies the mutual dependence of the two random variables. It has been widely used in computational neuroscience to analyze spiking neural systems, for example, characterizing the amount of information that the output spike trains carry about input signals. In the frequency domain, if the stimulus possesses Gaussian statistics, the estimate of MI rate can be expressed explicitly via the coherence function [43], [47], [48]

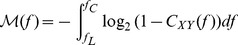
(9)where 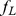 and 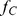 indicate the lower and upper cutoff frequencies of the stimulus, respectively.

## Supporting Information

Figure S1
**Coherence functions obtained from P-units with different P-values in response to narrowband RAM.** A compound signal with a narrow-band RAM, 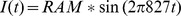, has been used to simulate the sensory signal generated in a group of fish (see [12]). Here a 70–120 Hz RAM was used to stimulate the P-unit models with different P-values (tuned by 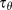 in Equation (7)). (A) The resulting maximum of coherence function between RAM and the P-unit's response, 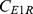, over 70–120 Hz increases with increasing P-values, while (B) the coherence function between envelope of RAM and the response, 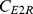, drops with increasing P-values. The result in (B) agrees with the experimental observation in [15].(TIF)

Text S1
**Matlab code to generate simulation signal,** 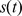 **, in** **Equation (1)** **.**
(PDF)
