# Supplementary figures and images for: Coding Conspecific Identity and Motion in the Electric Sense
Source: PLoS Comput Biol. 2012 Jul 12;8(7):e1002564. doi: 10.1371/journal.pcbi.1002564 (PMC3395610; doi:10.1371/journal.pcbi.1002564)

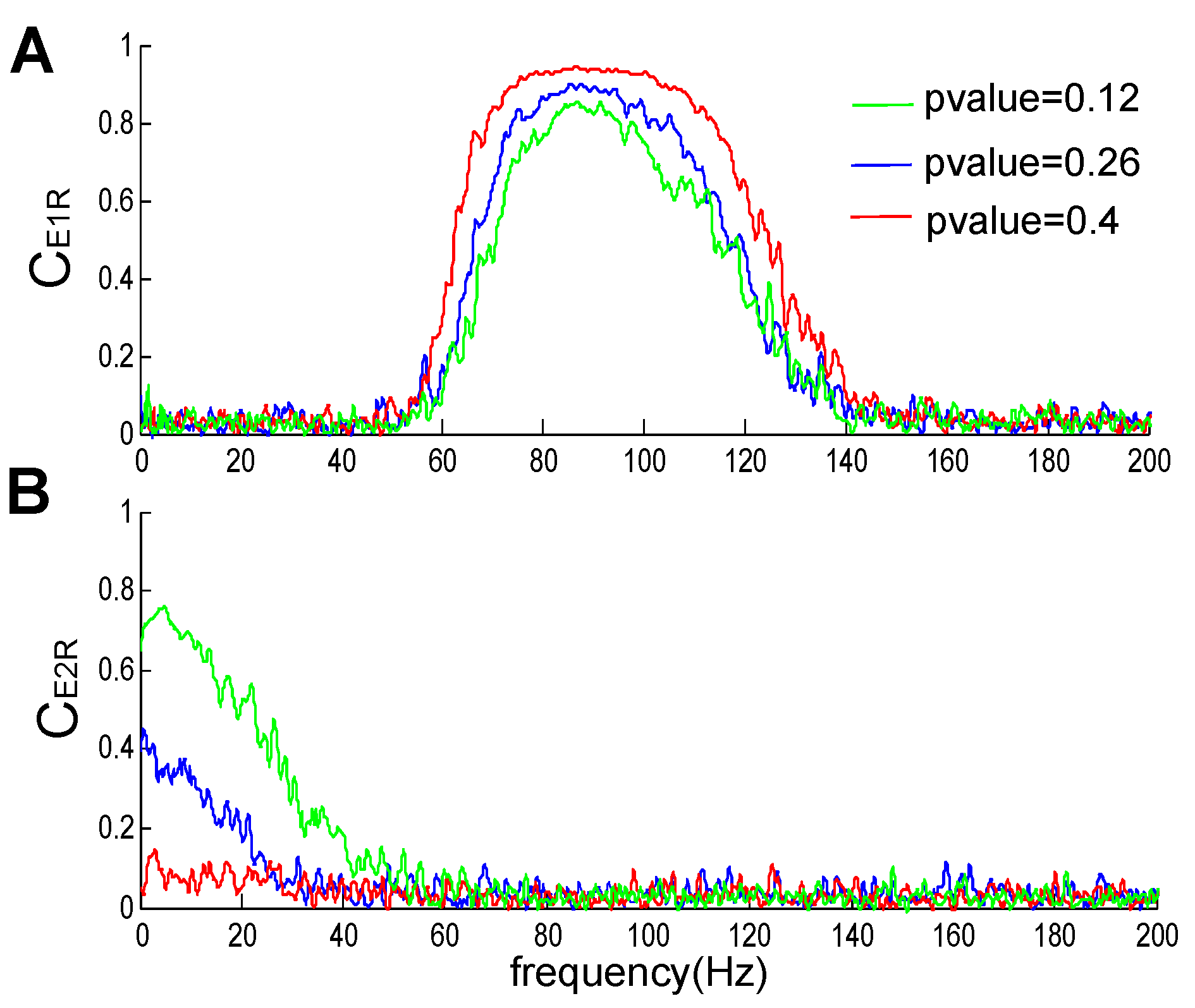

Supplement: Figure S1 — Coherence functions obtained from P-units with different P-values in response to narrowband RAM. A compound signal with a narrow-band RAM, , has been used to simulate the sensory signal generated in a group of fish (see [12]). Here a 70–120 Hz RAM was used to stimulate the P-unit models with different P-values (tuned by in Equation (7)). (A) The resulting maximum of coherence function between RAM and the P-unit's response, , over 70–120 Hz increases with increasing P-values, while (B) the coherence function between envelope of RAM and the response, , drops with increasing P-values. The result in (B) agrees with the experimental observation in [15]. (TIF) [file pcbi.1002564.s001.tif]
