# Supplementary material for: Coding Conspecific Identity and Motion in the Electric Sense
Source: PLoS Comput Biol. 2012 Jul 12;8(7):e1002564. doi: 10.1371/journal.pcbi.1002564 (PMC3395610; doi:10.1371/journal.pcbi.1002564)
Supplement: Text S1 — Matlab code to generate simulation signal, , in Equation (1) . (PDF) [file pcbi.1002564.s002.pdf]

Supporting Information - Text S1

for "Coding Conspecific Identity and Motion in the Electric Sense"

Na Yu, Ginette Hupé, Charles Garfinkle, John E Lewis, André Longtin

Matlab code to generate simulation signal, $s(t)$, in Equation (1)

```
% dt: timestep (in sec)
% N: length of simulation signal
% t: time
dt=0.00001;N=2^22;t=dt:dt:N*dt;

% eta: Ornstein-Uhlenbeck Process in Equation (3)
gamma=1;
rand('state',sum(100*clock));
xi=[0,randn(1,N-1)];
XI = cumsum(sqrt(2*gamma)*sqrt(dt)*xi);
XF = filter([0, gamma*dt], [1, -1+gamma*dt], XI);
OU= XF-XI;
eta=(OU-mean(OU))./std(OU);

% s: simulation signal in Equation (1)
f1=827;f2=763;A2=0.143;sigma2=0.08;
s=sin(2*pi*f1*t)+(A2+sigma2*eta).*sin(2*pi*f2*t);
```
